# Optimization of Elicitation Conditions to Enhance the Production of Potent Metabolite Withanolide from *Withania somnifera* (L.)

**DOI:** 10.3390/metabo12090854

**Published:** 2022-09-11

**Authors:** Manali Singh, Sanjeev Agrawal, Obaid Afzal, Abdulmalik S. A. Altamimi, Alya Redhwan, Nawaf Alshammari, Mitesh Patel, Mohd Adnan, Abdelbaset Mohamed Elasbali, Shahanavaj Khan

**Affiliations:** 1 Department of Biochemistry, C.B.S.H., G.B. Pant University of Agriculture and Technology, Pantnagar 263145, India; 2 Department of Biotechnology, Invertis University, Invertis Village, Bareilly- Lucknow National Highway, NH-24, Bareilly 243123, India; 3 Department of Pharmaceutical Chemistry, College of Pharmacy, Prince Sattam Bin Abdulaziz University, Al-Kharj 11942, Saudi Arabia; 4 Department of Health, College of Health and Rehabilitation Sciences, Princess Nourah bint Abdulrahman University, P.O. Box 84428, Riyadh 11671, Saudi Arabia; 5 Department of Biology, College of Science, University of Hail, Hail P.O. Box 2440, Saudi Arabia; 6 Department of Biotechnology, Parul Institute of Applied Sciences and Centre of Research for Development, Parole University, Vadodara 391760, India; 7 Department of Clinical Laboratory Science, College of Applied Science, Qurayyat, Jouf University, Sakaka 72341, Saudi Arabia; 8 Department of Medical Lab Technology, Indian Institute of Health and Technology (IIHT), Saharanpur 247554, India; 9 Department of Health Sciences, Novel Global Community Educational Foundation, Hebersham, NSW 2770, Australia

**Keywords:** chitosan, foliar spray, jasmonic acid, salicylic acid, *Withania somnifera*, withanolides, ashwagandha, thin-layer chromatography

## Abstract

This study aimed at optimizing conditions for increased withanolide production in *Withania somnifera*. The elicitors used for the foliar spray on the aerial parts of the plant were salicylic acid, jasmonic acid, and chitosan for the enhancement of withanolides in *Withania somnifera* under different environmental regimes. Three different elicitors, i.e., chitosan, jasmonic acid and salicylic acid, were applied on the plants through foliar route every 15th day for 6 months, and later plants were used for sample preparation. Further, the elicitors were used in different concentration, i.e., jasmonic acid (50, 200 and 400 ppm), chitosan (10, 50 and 100 ppm) and salicylic acid (0.5, 1 and 2 ppm). The elicitors were sprayed on the foliar parts of the plant between 10:00–11:00 a.m. on application days. For elicitor spray, a calibrated sprayer was used. The withanolide A/withaferin A was quantified through HPLC. It was found that in an open environment, maximum withaferin A content, i.e., 0.570 mg/g (DW), was recorded with jasmonic acid (50 ppm) treatment in comparison to control (0.067 mg/g DW). Thus, there was an 8.5-fold increase in the withaferin A content. Maximum withanolide A content of 0.352 mg/g (DW) was recorded when chitosan (50 ppm) was sprayed, while in the control, withanolide A content was recorded to be 0.031 mg/g (DW); thus, chitosan application increased the production of withanolide A by 11.3-fold. Under controlled conditions, maximum withaferin A content of 1.659 mg/g (DW) was recorded when plants were sprayed with chitosan (100 ppm), which was 8.1 times greater than the control content of 0.203 mg/g (DW). Maximum withanolide A content of 0.460 mg/g (DW) was recorded when chitosan (100 ppm) was applied, whereas in the control, withanolide A content was found to be 0.061 mg/g (DW). Thus, foliar spraying of elicitors in very low concentrations can serve as a low-cost, eco-friendly, labor-intensive and elegant alternative approach that can be practiced by farmers for the enhancement, consistent production and improved yield of withanolide A/withaferin A. This can be a suitable way to enhance plant productivity, thus increasing the availability of withanolide A and withaferin A for the health and pharma industry.

## 1. Introduction

Plants have long been known for producing chemical compounds known as secondary metabolites as a strategy to increase their self-defense. Secondary metabolites do not participate in essential metabolic roles. These metabolites are utilized in the pharmaceutical and chemical industries for manufacturing different types of medicines, important chemicals, and other allied products. However, a major issue with these plant-produced secondary metabolites is inconsistency in their amounts and quality. As a result of this, the pharma companies face a problem in drug formulations. Moreover, the wild plant varieties are uprooted for the extraction of the medicinally rich metabolites, resulting in loss of the elite germplasm. Thus, there is indeed a great requirement and interest in this area to increase the yield of secondary metabolites in medicinal plants by simple, cheap and easy methods so that these metabolites can be extracted easily and whole plants are not uprooted. Foliar application of elicitors is one way in which we can improve the quality of the plant’s secondary metabolites by increasing their biomass and yield [1].

*Withania somnifera* occupies a significant position in the Indian Ayurveda because of pharmacologically significant potent phytocompounds named withanolides [2,3]. Ashwagandha serves as an important source of herbal preparations for ayurvedic medical practitioners [4]. Ashwagandha is a priority medicinal herb that is used as a whole, or its different parts are used for the formulation of more than 100 traditional medicines. It has antioxidant, anti-inflammatory, anti-stress, anti-tumor, hemopoietic, and overall rejuvenating properties. Thus, *Withania somnifera* is widely cultivated in India for commercial pharmacological purposes and is highly demanded for export purposes [5].

Withanolides occur in almost the whole of the plant. The distinct types and quantities vary. The bioactive ingredients vary among the different species of *Withania somnifera* and environmental regimes. We reported earlier that withaferin A and withanolide A are found in higher quantities in different WS varieties and in varying concentrations [6]. Because of this, herbal formulations are not easily prepared [7]. With the alarming increase in the risks, cost, and side effects of chemically formulated drugs, people are now more inclined to rely on naturopathy. Naturopathy is progressively gaining worldwide significance for curing various diseases [8]. Most of the world’s population rely on naturopathy [9]. However, due to the inconsistency in the concentrations of bioactive metabolites, the preparation of herbal formulations is affected, and the growing commercial demand in the pharmaceutical industries cannot be met. Moreover, secondary metabolites are greatly influenced by the plant’s developmental and physiological stage and thus remain inconsistent in production [10]. Because of the lower amounts of the bioactive ingredients in wild plants, in vitro propagation of elite germplasm is usually carried out for their bioaccumulation [11]. However, their biosynthesis and bioaccumulation depend upon several factors [12].

Elicitors, namely jasmonic acid (JA) and methyl jasmonate (MeJA), have been known for increasing the production of secondary metabolites [11]. They have a significant impact in signal transduction processes. They play an important role in defense mechanisms in plants. These hormones have also been very effective in increasing production of secondary metabolites in in vitro plant cell cultures [12]. Jasmonic acid (JA), methyl jasmonate (MeJA) and salicylic acid (SA) enhance the production of secondary metabolites. Chitosan has been found to be effective in the accumulation of pharmaceutically important bioactive metabolites in in vitro cultures [13]. Moreover, the environmental factors also affect the accumulation of these secondary metabolites; thus, the biotic and abiotic factors are quite significant for increased withanolide A/withaferin A content. This research article focused on the optimization of elicitor concentrations under different conditions to enhance the withaferin A and withanolide A productivity of *Withania somnifera*.

## 2. Materials and Methods

### 2.1. Plant Material

One hundred 2-year-old *Withania somnifera* plants (variety, Jawahar-20) were purchased from CIMAP, Pantnagar, India. The ratio of soil and and vermicompost is an important factor. In the current study, the plants were grown in pots containing autoclaved soil and vermicompost in a 1:1 ratio; the soil chemical analysis is listed below in Table 1.

Fifty of the plants were kept in an open environment (mango garden), and 50 were grown in pots that were kept in a closed environment (transgenic polyhouse) maintained at 25 °C at G.B. Pant University of Agriculture and Technology, Pantnagar, Uttrakhand, India.

### 2.2. Application of Elicitors

Chitosan, jasmonic acid and salicylic acid were applied on the plants through the foliar route at a regular interval every 15th day for 6 months, and later, plants were used for sample preparation. Further, the elicitors were used in different concentrations, as follows: jasmonic acid, 50, 200 and 400 ppm; chitosan, 10, 50 and 100 ppm; and salicylic acid, 0.5, 1 and 2 ppm. Elicitors were sprayed between 10:00–11:00 a.m. on application days, while no elicitor sprays were applied to the control plants. The experiment was completed with three replicates of each set. For elicitor sprays, a calibrated sprayer was used. Withanolide A/withaferin A were quantified through HPLC [1]. The entire experimental setup was a randomized design in triplicate for the standardization of conditions for optimized enhancement of withanolide A and withaferin A contents and biomass production.

### 2.3. Preliminary Assessment for the Detection of Withanolide A and Withaferin A

To preliminarily test for the presence of withanolide A/withaferin A, the TLC was made of different samples (leaves, stem, and roots) of the Jawahar-20 variety of field-grown *Withania somnifera* plants.

### 2.4. Preparation of Methanolic Extracts

Fresh leaves, stems, and roots from the plant were taken. The tissue was subjected to drying in a hot air oven at 40 °C for 3–4 consecutive days until a constant dry weight was obtained. Then, the plant tissue was ground with the help of clean and dry mortar and pestle. One gram (dry weight) of powdered plant tissue was taken and percolated in 50 mL 80% methanol and sonicated for 20 min and placed on a rotary shaker at 30 °C at 100 rpm overnight. The resulting extracts were pooled and filtered through Whatman filter paper. Then, the methanolic extract was subjected to drying using a rotary vacuum evaporator maintained at 60 °C until completely dried residue was obtained. The dried residue was then dissolved in 1 mL methanol and was kept in vials in the refrigerator at 4 °C for later use in spotting the samples on the TLC plate. The experiment was conducted in triplicate.

Two types of TLC plates were used for the isolation of withanolide A and withaferin A: UV fluorescence and UV non-fluorescence plates. The UV fluorescence plates were Whatman silica gel-coated 250 µm, 20 × 20 cm, while UV non-fluorescence plates were Merck silica gel 60-coated 20 × 20 cm. Commercially available, pure withanolide A and withaferin A were used for preparing standards for HPLC. For preparation of standard stock solutions (1 mg/mL), withanolide A and withaferin A dried powder were dissolved in HPLC grade methanol. Later, from these stock solutions, a working solution was prepared. The mobile phase used for TLC was prepared by the method suggested by Sharma et al. 2007, with few changes, using toluene, ethyl acetate, and formic acid in the ratio of 5:5:1. Before TLC, the TLC glass chamber was pre-saturated with the mobile phase 30 min beforehand. The spray reagent used for TLC was prepared by protocol [14] with few modifications, using concentrated H_2_SO_4_, methanol, glacial acetic acid, and Anisaldehyde in the ratio of 5:85:10:0.5.

### 2.5. Spotting of Samples on TLC Plates

A TLC plate of 20 × 20 cm was taken and with the help of a fine, sharp-pointed pencil 1 cm from below, a line was drawn. On this line, equidistant spots of different samples were placed with the help of a graduated capillary tube (10 µL). After placing each spot, the plate was air-dried to avoid the spreading of the spots. After drying, the plate was carefully placed in the TLC glass chamber, and the solvent was allowed to run by capillary action. When the solvent was 1 cm away from reaching the end of the plate, the plate was taken out and then dried with the help of a hair drier. Then, the spray reagent was sprayed with the help of a sprayer to visualize the bands and compare with standard withaferin A and withanolide A (the bands were visualized under UV light for UV-sensitive TLC plates).

### 2.6. Quantification of Withanolide A and Withaferin A

Withanolide A/Withaferin A contents were quantified as described earlier [15,16]. HPLC (C18 reverse phase column) samples were prepared from different tissues (i.e., leaves, stem, and roots) of Jawahar-20 to assess the withanolides contents. The bioactive compounds were expressed as mg per gram dry weight (mg/DW).

Fresh leaves, stems, and roots from the plant were used. The plant tissue was subjected to drying in a hot air oven at 40 °C for 3–4 consecutive days until a constant dry weight was obtained. Then, the plant tissue was ground with the help of clean and dry mortar and pestle. One gram (dry weight) of powdered plant tissue was percolated in 50 mL 80% methanol, then sonicated for 20 min and placed on a rotary shaker at 30 °C at 100 rpm overnight. The experiment was conducted in triplicate. Methanolic extracts thus obtained were pooled to make a single composite sample and filtered through Whatman filter paper. Then, the methanolic extract was subjected to drying using a rotary vacuum evaporator maintained at 60 °C until completely dried residue was obtained. The dried residue was re-dissolved in HPLC grade methanol (4 mL). Further, the sample was decolorized using charcoal and centrifuged at 8000 rpm for 15 min. Finally, the supernatant was filtered and stored at 4 °C until further use. Methanol and water (HPLC grade) were used in the ratio of 70:30. The mixture was filtered through nylon filter membranes (0.45 µ). The solvent was used for HPLC after water sonication.

Quantification of withanolides was performed using reverse HPLC (Agilent 1120 Compact LC) with a 5 µm (ODS34.6 × 250 mm) C18 column [6]. The flow rate of the solvent was maintained at 1 mL min^−1^ and temperature at 30 °C. The UV detector was used for the quantification of withanolides with a set wavelength of 254 nm.

Further, a 10 µL sample volume was injected. The retention time and peak area were recorded. Then, withanolide A and withaferin A contents were calculated with the help of a standard curve [1].

## 3. Results

**Figure 1:**
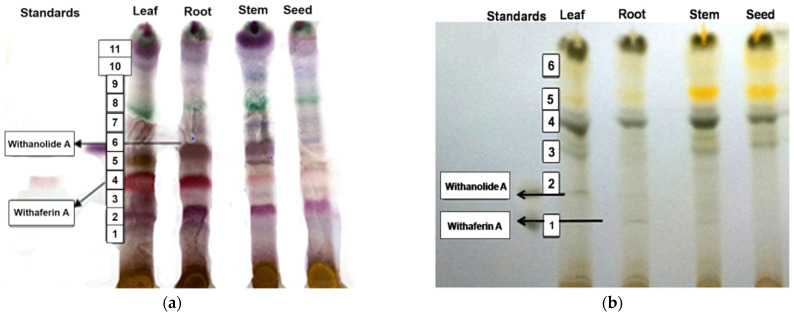
TLC chromatogram of (**a**) charcoal treated sample and (**b**) charcoal untreated sample.

To isolate withaferin A and withanolide A, the TLC profiles were obtained from the methanolic extracts of leaves, stems and roots of WS Jawahar-20. The spots of the sample extracts were visualized and identified with the help of the Rf value of the standard withanolide A (Rf = 0.50) and standard withaferin A (Rf = 0.41) (Table 2). TLC of the charcoal treated sample did not show very clear bands of withaferin A or withanolide A, while very clear bands of withaferin A and withanolide A were observed with the non-charcoal treated leaf samples of Jawahar-20 (Figure 1a,b). Moreover, from the intensity of bands, it was concluded that withaferin A and withanolide A were present in higher amounts in leaf, followed by roots and stems, of Jawahar-20. Based on the bands of Rf values for standard withaferin A and withanolide A, the contents of withaferin A and withanolide A in the different tissues were analyzed qualitatively (Table 2). Apart from the bands for withaferin A and withanolide A, several other bands were also detected in the chromatogram but could not be identified (Figure 1).

### 3.1. Effect of Elicitors on the Accumulation of Withanolide A and Withaferin A Contents

HPLC peak profiles revealed that the peak for standard withanolide A was found at the retention time of 6.2 min, whereas the peak for standard withaferin A was found at the retention time of 5 min, which was further confirmed after obtaining peaks for the reference standards at the same retention times (Table 2).

**Figure 2:**
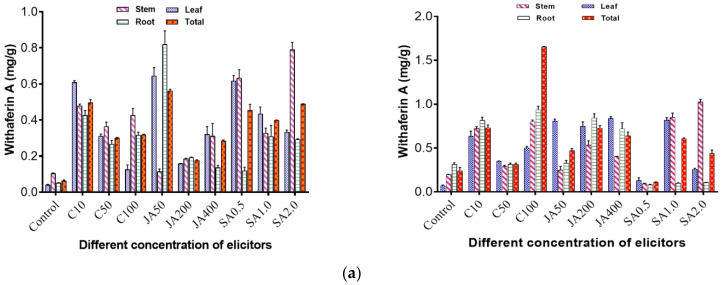

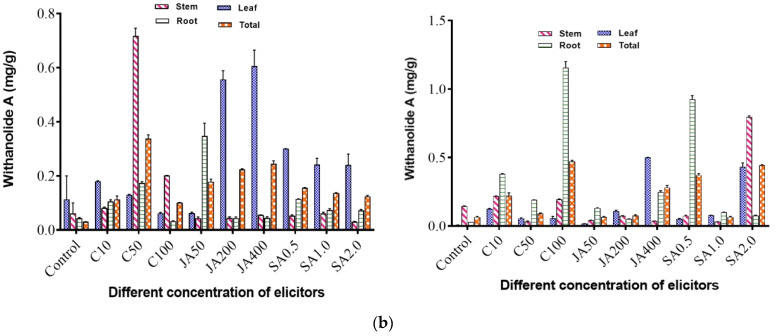
(**a**) Effect of elicitors, namely chitosan, jasmonic acid and salicylic acid, on withaferin A content in different tissues such as leaf, stem, and root of WS in open and controlled environments. Total is the average of three tissues (leaf, stem and root); C10, C50, C100 = concentration of chitosan at 10 ppm, 50 ppm and 100 ppm; JA 50, JA 200, JA 400 = concentration of jasmonic acid at 50 ppm, 200 ppm and 400 ppm; SA 0.5, SA 1.0, SA 2.0 = concentration of salicylic acid at 0.5 ppm, 1 ppm and 2 ppm. (**b**) Effect of elicitors, namely chitosan, jasmonic acid and salicylic acid, on withanolide A content in different tissues such as leaf, stem, and root of WS in open and controlled environments.

It was observed that both open and controlled environmental conditions affected the withanolide A and withaferin A contents. In the open environment, maximum withaferin A content of 0.570 mg/g (DW) was obtained when jasmonic acid (50 mg/L) was applied to the plants. The implication is that jasmonic acid promoted a high production yield of withaferin A. This was followed by 0.513 mg/g (DW) when chitosan (10 mg/L) was applied, whereas with the control, withaferin A of 0.067 mg/g (DW) was obtained (Figure 2a). The maximum withanolide A content of 0.352 mg/g (DW) was obtained when chitosan (50 mg/L) was applied to the plants, followed by 0.256 mg/g when jasmonic acid (400 mg/L) was applied, whereas in the control, withanolide A was found to be 0.031 mg/g (DW) (Figure 2b).

On the other hand, in the controlled environment, a maximum 1.659 mg/g (DW) of withaferin A was obtained when chitosan (100 mg/L) was applied to the plants. This was followed by a yield of 0.756 mg/g with the application of jasmonic acid (200 mg/L), while in the control, withaferin A content was found to be 0.203 mg/g (DW) (Figure 2a). For withanolide A, maximum content of 0.460 mg/g (DW) was obtained when chitosan (100 mg/L) was applied, followed by 0.447 mg/g (DW) when salicylic acid (2 mg/L) was applied to the plants, whereas in the control, 0.061 mg/g (DW) of withanolide A was obtained (Figure 2b).

### 3.2. Effect of Elicitors on the Accumulation of Biomass and Yield of Withanolide A and Withaferin A

**Figure 3:**
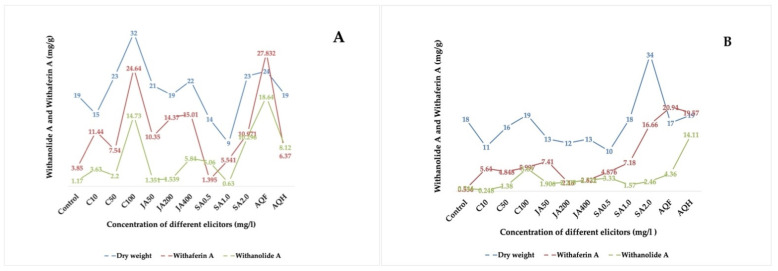
Effect of chitosan, jasmonic acid and salicylic acid on plant biomass and yield of withaferin A and withanolide A in WS under open environment (**A**) and controlled environment (**B**). C10, C50, C100 = concentration of chitosan at 10 ppm, 50 ppm and 100 ppm; JA 50, JA 200, JA 400 = concentration of jasmonic acid at 50 ppm, 200 ppm and 400 ppm; SA 0.5, SA 1.0, SA 2.0 = concentration of salicylic acid at 0.5 ppm, 1 ppm and 2 ppm.

In the present study, it was found that the foliar application of elicitors not only increased the plant biomass but also resulted in increased accumulation of withanolide A and withaferin A contents (Table 3 and Figure 3). However, no correlation was observed between the production of withaferin A and withanolide A.

## 4. Discussion

Plant metabolites are used in the pharmaceutical industries and chemical industries for the production of various types of drugs, chemicals, and other related products. However, an important issue with these plant-derived metabolites is the inconsistency in their amounts and quality. The crude extracts of *Withania somnifera* contain various types of pharmacologically active phytochemicals [17]. The primary alkaloids extracted from the different parts of the plant mainly comprise withanolides, widely known for their medicinal properties [18,19,20,21,22]. The major biochemical constituents of withania root are steroidal lactones in a class of phytoconstituents known as withanolides [23]. To date, up to 19 withanolide derivatives have been isolated from withania roots [24]. Withaferin A and withanolide A have been found to be predominant in the varietal distribution of *Withania somnifera* [25].

A quantitative analysis was performed on WS by Ray and Jha using TLC densitometry that revealed the high percentage of withaferin A content in leaf samples [26,27] by visualizing the spots and comparing them with the standards for withaferin A (Rf = 0.34) and withanolide A (Rf = 0.51). TLC densitometry showed the presence of withaferin A in leaves (1.6%) [28]. Quantification of withaferin A from in vitro grown samples has also been reported [29].

In a study [29], it was found that withaferin A content was higher in the leaves than in other parts of the same plants. In Jawahar-20 and Poshita (in vitro and seed propagated) plants, it was reported that withaferin A content was higher in the leaves in comparison to roots [30]. Stem material contained the lowest amount of total withanolide (withaferin A and withanolide A) [31]. In roots, withanolide A was predominant in all chemotypes [32].

The production and accumulation of these secondary metabolites is species- as well as chemotype-specific strictly under the spatial and temporal regulation of gene expression [33]. In previous studies, it has been reported that the chemotypic variation depends not only on the developmental stages and tissue but also on the geographical locations and seasonal variations [34].

However, the variations in phytochemical constituents endanger the compositional standardization and preparation of herbal formulations for commercial purposes [35]. It has been reported that selection of the best variety is necessary [36,37,38]. The use of elicitors for the modification of metabolite yields has emerged recently for increased accumulation of secondary metabolites in WS [39,40]. Previous studies have reported the maximal expression of withanolide biosynthetic genes in young leaves treated with methyl jasmonate (MJ) and salicylic acid (SA) [41,42]. Crude extracts of WS contain various types of pharmacologically active phytochemicals that are reported to be species- as well as chemotype-specific [43]. Further, hairy root cultures of *P. indica* have shown enhanced production of the bioactive compound plumbagin in response to jasmonic acid (JA) treatment [44].

Pre-harvest exogenous application of MeJ and chitosan was found to induce lycopene production in tomato plants [45]. Chitosan increases withanolides accumulation in the adventitious root of WS [46]. It was also reported that chitosan enhanced the withanolide concentration in cell suspension cultures [47]. It was found that chitosan also induced the production of varied plant secondary metabolites in in vitro cell cultures [48]. It has been reported that optimization of elicitation conditions with methyl jasmonate and salicylic acid improved the production of withanolides in the adventitious root culture of *Withania somnifera* [49]. Further, the elicitor concentration, specificity, time of exposure, and culture conditions have been shown to be crucial factors for the elicitation process [50].

Different elicitors in different concentrations were found to produce withanolide A and withaferin A differentially. Environmental conditions had very little or no effect on plant biomass. However, a significant enhancement in the yield of withanolide A and withaferin A were observed in the controlled environment relative to the open environment. Environmental conditions modulate the accumulation of withanolides in WS [6]. However, efforts to increase withanolide production have faced many challenges due to several environmental factors such as humidity, rainfall, temperature, etc. [51].

## 5. Conclusions

*Withania somnifera* has proven to be important in the pharmaceutical industries due to the availability of varied bioactive compounds (withanolides). Elicitors were found to be effective in promoting the enhanced accumulation of withanolide A and withaferin A. The elicitors also increased the plant biomass. Moreover, even slightly higher concentrations of elicitors were not found to be toxic for plant growth or users. Significant increases in the yields of withanolide A and withaferin A were observed in the controlled environment compared to the open environment. Thus, foliar spraying of elicitors in very low concentrations can serve as a low-cost, eco-friendly, labor-intensive and elegant alternative approach that can be practiced by farmers for the enhancement, consistent production and better yield of withanolide A/withaferin A. Foliar spraying of elicitors can be a suitable way to enhance plant productivity. It is also an easy and cheap method for farmers. This method can be very helpful in order to increase plant productivity and meet the increasing demand in the pharma industries.

## Figures and Tables

**Table 1:** Element analysis of soils used in elicitation study for potted plants grown in mango garden and transgenic polyhouse.

Soil Samples	Control	Mango Garden	Transgenic Laboratory
Salinity (PSU)	33	44	94
Electrical conductivity (µS/cm)	65	56	187
pH (With pH Scale)	7.23	7.40	7.86
Temperature (°C)	29	28.5	25
Organic carbon (mg/L)	12	6.3	4.3
Potassium (K) (mg/g)	18.3	22.6	69.9
Phosphorous (mg/g)	0.04	0.035	0.032
Nitrogen (N_2_) (mg/g)	3.3	3.8	4.2
Vanadium (μg/g)	112.35	117.96	121.96
Chromium (μg/g)	79.06	65.03	69.50
Manganese (μg/g)	452.56	402	418.03
Iron (μg/g)	52,063.91	50,569.36	45,235.25
Cobalt (μg/g)	62.36	36.25	30.24
Nickel (μg/g)	7.45	6.98	56.32
Copper (μg/g)	8.65	75.25	60.24
Zinc (μg/g)	39.57	48.27	52.45

**Table 2:** Screening of different varieties of WS for withaferin A and withanolide A.

Genotypes/Standard	Rf of Withaferin A	Rf of Withanolide A
Withaferin A Standard	0.41	-
Withanolide A Standard	-	0.50
Jawahar-20	-	-
Leaf	0.41 (+++)	0.50 (+++)
Root	0.41 (++)	0.50 (+)
Stem	0.41 (++)	0.41 (++)

**Table 3:** Effect of elicitors (chitosan, jasmonic acid and salicylic acid) on plant biomass and yield of withaferin A and withanolide A in WS in open and controlled environments. C10, C50, C100 = concentration of chitosan at 10 ppm, 50 ppm and 100 ppm; JA 50, JA 200, JA 400 = concentration of jasmonic acid at 50 ppm, 200 ppm and 400 ppm; SA 0.5, SA 1.0, SA 2.0 = concentration of salicylic acid at 0.5 ppm, 1 ppm and 2 ppm.

Elicitors	Open Environment					Controlled Environment				
	Plant Height (cm)	Plant Biomass FW (g)	Plant Biomass DW (g)	Yield (Withaferin A) mg/Plant	Yield (Withanolide A) mg/Plant	Plant Height (cm)	Plant Biomass FW (g)	Plant Biomass DW (g)	Yield (Withaferin A) mg/Plant	Yield (Withanolide A) mg/Plant
Control	30 ± 0.01 ^a^	27 ± 0.2 ^ab^	18 ± 0.3 ^ab^	0.536 ^a^	0.248 ^a^	30 ± 0.15 ^a^	25 ± 0.6 ^b^	19 ± 0.2 ^b^	3.85 ^b^	1.17 ^a^
C10	45 ± 0.12 ^g^	32 ± 0.0 ^bc^	11 ± 0.5 ^a^	5.64 ^b^	1.38 ^b^	33 ± 0.2 ^b^	22 ± 0.0 ^a^	15 ± 0.1 ^g^	11.445 ^g^	3.62 ^d^
C50	57 ± 0.27 ^i^	42 ± 0.1 ^ab^	16 ± 0.4 ^bc^	4.848 ^f^	5.63 ^h^	61 ± 0.01 ^g^	39 ± 0.2 ^f^	23 ± 0.1 ^f^	7.544 ^e^	2.200 ^c^
C100	53 ± 0.55 ^g^	38 ± 0.5 ^cd^	19 ± 0.01 ^bc^	5.997 ^d^	1.906 ^c^	72 ± 0.3 ^h^	45 ± 0.2 ^g^	32 ± 0.0 ^j^	24.64 ^h^	14.73 ^i^
JA50	55 ± 0.36 ^h^	40 ± 0.11 ^cd^	13 ± 0.01 ^ab^	7.41 ^e^	2.205 ^c^	61 ± 0.1 ^g^	34 ± 0.7 ^d^	21 ± 0.2 ^f^	10.35 ^f^	1.351 ^a^
JA200	57 ± 0.35 ^i^	41 ± 0.14 ^bc^	12 ± 0.8 ^ab^	2.16 ^e^	2.73 ^e^	41 ± 0.1 ^e^	29 ± 0.1 ^c^	19 ± 0.2 ^h^	14.37 ^h^	1.539 ^b^
JA400	50 ± 0.78 ^f^	36 ± 0.23 ^cd^	13 ± 0.1 ^ab^	2.822 ^c^	3.33 ^f^	50 ± 0.0 ^f^	36 ± 0.6 ^e^	22 ± 0.5 ^i^	15.01 ^i^	5.84 ^f^
SA0.5	47 ± 0.65 ^e^	35 ± 0.1 ^cd^	10 ± 0.1 ^ab^	4.876 ^c^	1.57 ^b^	38 ± 0.5 ^c^	24 ± 0.2 ^b^	14 ± 0.1 ^a^	1.395 ^a^	5.06 ^f^
SA1.0	37 ± 0.12 ^b^	29 ± 0.01 ^bc^	18 ± 0.08 ^e^	7.182 ^a^	2.46 ^d^	32 ± 0.25 ^b^	23 ± 0.4 ^a^	9 ± 0.6 ^c^	5.541 ^c^	0.63 ^a^
SA2.0	50 ± 0.06 ^f^	40 ± 0.1 ^d^	34 ± 0.12 ^c^	16.66 ^de^	4.36 ^g^	40 ± 0.8 ^d^	38 ± 0.5 ^f^	23 ± 0.8 ^fg^	10.971 ^fg^	10.296 ^h^
CD at 1%	2.06	2.5	3.29	3.30	1.17	2.41	2.15	1.9	1.77	0.02
CD at 5%	1.4	1.83	2.35	2.36	0.83	1.72	1.25	1.3	1.26	0.02
CV	1.4	2.44	4.64	4.47	3.62	5.2	3.5	4.8	3.23	2.88

Data shown are mean ± SE_m_ (*n* = 3). The genotypes with same superscript within each assay (parameter) are not significantly different at *p* ≤ 0.05, according to Duncan multiple comparison procedure (ANOVA).

## Data Availability

All data is available in the manuscript.

## References

[1] Singh M., Poddar N.K., Singh D., Agrawal S. (2020). Foliar Application of Elicitors Enhanced the Yield of Withanolide Contents in *Withania somnifera* (L.) Dunal (Variety, Poshita). 3 Biotech.

[2] Kushwaha S., Roy S., Maity R., Mallick A., Soni V.K., Singh P.K., Chaurasiya N.D., Sangwan R.S., Misra-Bhattacharya S., Mandal C. (2012). Chemotypical Variations in Withania somnifera Lead to Differentially Modulated Immune Response in BALB/c Mice. Vaccine.

[3] Yang E.S., Choi M.J., Kim J.H., Choi K.S., Kwon T.K. (2011). Withaferin A Enhances Radiation-Induced Apoptosis in Caki Cells through Induction of Reactive Oxygen Species, Bcl-2 Downregulation and Akt Inhibition. Chem. Biol. Interact..

[4] Sharma L.K., Madina B.R., Chaturvedi P., Sangwan R.S., Tuli R. (2007). Molecular Cloning and Characterization of One Member of 3β-Hydroxy Sterol Glucosyltransferase Gene Family in Withania somnifera. Arch. Biochem. Biophys..

[5] Hassannia B., Logie E., Vandenabeele P., Berghe T.V., Berghe W.V. (2020). Withaferin A: From Ayurvedic Folk Medicine to Preclinical Anti-Cancer Drug. Biochem. Pharmacol..

[6] Singh M., Shah P., Punetha H., Agrawal S. (2018). Varietal Comparison of Withanolide Contents in Different Tissues of *Withania somnifera* (L.) Dunal (Ashwagandha). Int. J. Life Sci. Sci. Res..

[7] Sangwan R.S., Chaurasiya N.D., Misra L.N., Lal P., Uniyal G.C., Sharma R., Sangwan N.S., Suri K.A., Qazi G.N., Tuli R. (2004). Phytochemical Variability in Commercial Herbal Products and Preparations of Withania somnifera (Ashwagandha). Curr. Sci..

[8] Kuo Y.-T., Liao H.-H., Chiang J.-H., Wu M.-Y., Chen B.-C., Chang C.-M., Yeh M.-H., Chang T.-T., Sun M.-F., Yeh C.-C. (2018). Complementary Chinese Herbal Medicine Therapy Improves Survival of Patients with Pancreatic Cancer in Taiwan: A Nationwide Population-Based Cohort Study. Integr. Cancer Ther..

[9] Renu S., Manvi M., Sapna B. (2010). Evaluation of Antibacterial Potential of Stem Bark of Moringa Oleifera Lam. Bioscan.

[10] Thakur G.S., Sharma R., Sanodiya B.S., Baghel R., Thakur R., Singh B.N., Savita A., Dubey A., Sikarwar L., Jaiswal P. (2013). In Vitro Induction of Tuber Formation for the Synthesis of Secondary Metabolites in Chlorophytum Borivilianum Sant. et Fernand. Afr. J. Biotechnol..

[11] Ali M., Abbasi B.H., Ali G.S. (2015). Elicitation of Antioxidant Secondary Metabolites with Jasmonates and Gibberellic Acid in Cell Suspension Cultures of *Artemisia Absinthium* L.. Plant Cell Tissue Organ. Cult..

[12] Zulak K.G., Cornish A., Daskalchuk T.E., Deyholos M.K., Goodenowe D.B., Gordon P.M.K., Klassen D., Pelcher L.E., Sensen C.W., Facchini P.J. (2007). Gene Transcript and Metabolite Profiling of Elicitor-Induced Opium Poppy Cell Cultures Reveals the Coordinate Regulation of Primary and Secondary Metabolism. Planta.

[13] Ferri M., Tassoni A. (2011). Chitosan as Elicitor of Health Beneficial Secondary Metabolites in in Vitro Plant Cell Cultures. Handb. Chitosan Res. Appl. Nov. Sci. Publ. N. Y..

[14] Senthil K., Thirugnanasambantham P., Oh T.J., Kim S.H., Choi H.K. (2015). Free Radical Scavenging Activity and Comparative Metabolic Profiling of in Vitro Cultured and Field Grown Withania somnifera Roots. PLoS ONE.

[15] Gupta A., Ansari S., Gupta S., Narwani M., Gupta M., Singh M. (2019). Therapeutics Role of Neem and Its Bioactive Constituents in Disease Prevention and Treatment. J. Pharmacogn. Phytochem..

[16] Singh P., Guleri R., Angurala A., Kaur K., Kaur K., Kaul S.C., Wadhwa R., Pati P.K. (2017). Addressing Challenges to Enhance the Bioactives of Withania somnifera through Organ, Tissue, and Cell Culture Based Approaches. Biomed Res. Int..

[17] Chatterjee S., Srivastava S., Khalid A., Singh N., Sangwan R.S., Sidhu O.P., Roy R., Khetrapal C.L., Tuli R. (2010). Comprehensive Metabolic Fingerprinting of Withania somnifera Leaf and Root Extracts. Phytochemistry.

[18] Lee J., Hahm E.-R., Singh S. (2010). V Withaferin A Inhibits Activation of Signal Transducer and Activator of Transcription 3 in Human Breast Cancer Cells. Carcinogenesis.

[19] Maitra R., Porter M.A., Huang S., Gilmour B.P. (2009). Inhibition of NFκB by the Natural Product Withaferin A in Cellular Models of Cystic Fibrosis Inflammation. J. Inflamm..

[20] Mayola E., Gallerne C., Esposti D.D., Martel C., Pervaiz S., Larue L., Debuire B., Lemoine A., Brenner C., Lemaire C. (2011). Withaferin A Induces Apoptosis in Human Melanoma Cells through Generation of Reactive Oxygen Species and Down-Regulation of Bcl-2. Apoptosis.

[21] Min K., Choi K., Kwon T.K. (2011). Withaferin A Down-Regulates Lipopolysaccharide-Induced Cyclooxygenase-2 Expression and PGE2 Production through the Inhibition of STAT1/3 Activation in Microglial Cells. Int. Immunopharmacol..

[22] Mohan R., Hammers H., Bargagna-Mohan P., Zhan X., Herbstritt C., Ruiz A., Zhang L., Hanson A., Conner B., Rougas J. (2004). Withaferin A Is a Potent Inhibitor of Angiogenesis. Angiogenesis.

[23] Ganzera M., Choudhary M.I., Khan I.A. (2003). Quantitative HPLC Analysis of Withanolides in Withania somnifera. Fitoterapia.

[24] Zhao J., Nakamura N., Hattori M., Kuboyama T., Tohda C., Komatsu K. (2002). Withanolide Derivatives from the Roots of Withania somnifera and Their Neurite Outgrowth Activities. Chem. Pharm. Bull..

[25] Thirugnanasambantham P., Senthil K., Oh T.J., Choi H.-K. (2015). Comparative Chemometric Profiles between Leaf Tissues of Withania somnifera Cultured in Vitro and Field. Int. J. Pharm. Pharm. Sci..

[26] Gupta A.P. (1996). Quantitative Determination of Withanferin-A in Different Plant Parts of Withania somnifera by TLC Densitometry. J. Medi. Aro. Plant Sci..

[27] Ray S., Jha S. (1999). Withanolide Synthesis in Cultures of Withania somnifera Transformed with Agrobacterium Tumefaciens. Plant Sci..

[28] Mir B.A., Khazir J., Hakeem K.R., Koul S., Cowan D.A. (2014). Enhanced Production of Withaferin-A in Shoot Cultures of *Withania somnifera* (L.) Dunal. J. Plant Biochem. Biotechnol..

[29] Siriwardane A.S., Dharmadasa R.M., Samarasinghe K. (2013). Distribution of Withaferin A, an Anticancer Potential Agent, in Different Parts of Two Varieties of *Withania somnifera* (L.) Dunal. Grown in Sri Lanka. Pakistan J. Biol. Sci..

[30] Das A., Kumar D.A. (2011). Assessment of Cytomorphological Parameters and Chemical Contents in in Vitro and Seed Propagated Plants of Elite Genotypes of *Withania somnifera* (L.) Dunal. Int. J. Res. Ayurveda Pharm..

[31] Dewir Y.H., Chakrabarty D., Lee S.-H., Hahn E.-J., Paek K.-Y. (2010). Indirect Regeneration of Withania somnifera and Comparative Analysis of Withanolides in in Vitro and Greenhouse Grown Plants. Biol. Plant..

[32] Gupta S.M., Pandey P., Grover A., Patade V.Y., Singh S., Ahmed Z. (2013). Cloning and Characterization of GPAT Gene from Lepidium Latifolium L.: A Step towards Translational Research in Agri-Genomics for Food and Fuel. Mol. Biol. Rep..

[33] Pathak R.K., Taj G., Pandey D., Arora S., Kumar A. (2013). Modeling of the MAPK Machinery Activation in Response to Various Abiotic and Biotic Stresses in Plants by a System Biology Approach. Bioinformation.

[34] Chaurasiya N.D., Sangwan N.S., Sabir F., Misra L., Sangwan R.S. (2012). Withanolide Biosynthesis Recruits Both Mevalonate and DOXP Pathways of Isoprenogenesis in Ashwagandha Withania somnifera L.(Dunal). Plant Cell Rep..

[35] Viji M.O., Mathew M.M., Parvatham R. (2013). Effects of Light Intensity and Imbibition Frequency of in Vivo and in Vitro Propagated Seeds of *Withania somnifera* (L.) Poshita on Germination. Int. J. Curr. Microbiol. Appl. Sci..

[36] Dhar R.S., Verma V., Suri K.A., Sangwan R.S., Satti N.K., Kumar A., Tuli R., Qazi G.N. (2006). Phytochemical and Genetic Analysis in Selected Chemotypes of Withania somnifera. Phytochemistry.

[37] Kumar A., Mir B.A., Sehgal D., Dar T.H., Koul S., Kaul M.K., Raina S.N., Qazi G.N. (2011). Utility of a Multidisciplinary Approach for Genome Diagnostics of Cultivated and Wild Germplasm Resources of Medicinal Withania somnifera, and the Status of New Species, W. Ashwagandha, in the Cultivated Taxon. Plant Syst. Evol..

[38] Scartezzini P., Antognoni F., Conte L., Maxia A., Troia A., Poli F. (2007). Genetic and Phytochemical Difference between Some Indian and Italian Plants of *Withania somnifera* (L.) Dunal. Nat. Prod. Res..

[39] Singh N., Iqbal Z., Ansari T.A., Khan M.A., Ali N., Khan A., Singh M. (2019). The Portent Plant with a Purpose: Aloe Vera. J. Pharmacogn. Phytochem..

[40] Sivanandhan G., Kapil Dev G., Jeyaraj M., Rajesh M., Muthuselvam M., Selvaraj N., Manickavasagam M., Ganapathi A. (2013). A Promising Approach on Biomass Accumulation and Withanolides Production in Cell Suspension Culture of *Withania somnifera* (L.) Dunal. Protoplasma.

[41] Agarwal A.V., Gupta P., Singh D., Dhar Y.V., Chandra D., Trivedi P.K. (2017). Comprehensive Assessment of the Genes Involved in Withanolide Biosynthesis from Withania somnifera: Chemotype-Specific and Elicitor-Responsive Expression. Funct. Integr. Genomics.

[42] Singh A., Singh P.K. (2008). Salicylic Acid Induced Biochemical Changes in Cucumber Cotyledons. Indian J. Agric. Biochem..

[43] Bhasin S., Singh M., Singh D. (2019). Review on Bioactive Metabolites of *Withania somnifera* (L.) Dunal and Its Pharmacological Significance. J. Pharmacogn. Phytochem..

[44] Gangopadhyay M., Dewanjee S., Bhattacharya S. (2011). Enhanced Plumbagin Production in Elicited Plumbago Indica Hairy Root Cultures. J. Biosci. Bioeng..

[45] Osano A., Fultang N., Davis J. (2017). Exogenous Pre-Harvest Treatment with Methyl Jasmonate and Chitosan Elicits Lycopene Biosynthesis in Tomato Plants. J Env. Sci Eng.

[46] Sivanandhan G., Arun M., Mayavan S., Rajesh M., Mariashibu T.S., Manickavasagam M., Selvaraj N., Ganapathi A. (2012). Chitosan Enhances Withanolides Production in Adventitious Root Cultures of *Withania somnifera* (L.) Dunal. Ind. Crops Prod..

[47] Sivanandhan G., Selvaraj N., Ganapathi A., Manickavasagam M. (2014). Enhanced Biosynthesis of Withanolides by Elicitation and Precursor Feeding in Cell Suspension Culture of *Withania somnifera* (L.) Dunal in Shake-Flask Culture and Bioreactor. PLoS ONE.

[48] Dhar N., Rana S., Bhat W.W., Razdan S., Pandith S.A., Khan S., Dutt P., Dhar R.S., Vaishnavi S., Vishwakarma R. (2013). Dynamics of Withanolide Biosynthesis in Relation to Temporal Expression Pattern of Metabolic Genes in *Withania somnifera* (L.) Dunal: A Comparative Study in Two Morpho-Chemovariants. Mol. Biol. Rep..

[49] Dhar N., Rana S., Razdan S., Bhat W.W., Hussain A., Dhar R.S., Vaishnavi S., Hamid A., Vishwakarma R., Lattoo S.K. (2014). Cloning and Functional Characterization of Three Branch Point Oxidosqualene Cyclases from *Withania somnifera* (L.) Dunal. J. Biol. Chem..

[50] Vasconsuelo A., Boland R. (2007). Molecular Aspects of the Early Stages of Elicitation of Secondary Metabolites in Plants. Plant Sci..

[51] Singh M., Shah P., Punetha H., Gaur A.K., Kumar A., Agrawal S. (2017). Isolation and Quantification of a Potent Anti Cancerous Compound, Withaferin A from the Aerial Parts of Withania somnifera (Ashwagandha). Ad Plant Sci.

